# Healthcare professionals’ perspectives on the utility of chronic postsurgical pain prediction profiles in perioperative care: a qualitative study

**DOI:** 10.1186/s44158-025-00332-0

**Published:** 2026-01-09

**Authors:** Cecilie Merethe Øvrebotten, Runar Tengel Hovland, Signe Berit Bentsen, Hans Jacob Vøllestad Westbye, Christian Moltu

**Affiliations:** 1 Department of Surgery, District General Hospital of Førde, Førde, Norway; 2 Department of Psychiatry, District General Hospital of Førde, Førde, Norway; 3 Department of Research and Innovation, District General Hospital of Førde, Førde, Norway; 4 Department of Health and Caring Sciences, Western Norway University of Applied Sciences, Førde, Norway; 5 Department of Health and Caring Sciences, Western Norway University of Applied Sciences, Haugesund, Norway; 6 Division of Emergency and Critical Care, Oslo University Hospital, Oslo, Norway

**Keywords:** Chronic postsurgical pain, Focus groups, Health personnel, Machine learning, Prediction methods, Machine, Prediction model, Risk prediction, Qualitative research

## Abstract

**Background:**

Chronic postsurgical pain affects surgical patients with a mean incidence of approximately 20%, posing a major public health concern with substantial implications for patients and healthcare systems. Despite increasing knowledge of risk factors, the incidence of chronic postsurgical pain remains high. Hence, there is growing interest in developing individualised pain management strategies using predictive risk. A novel chronic postsurgical pain risk assessment system using machine learning is under development in Western Norway. As a first step in implementing the risk assessment system, this study explored how in-hospital healthcare professionals perceive the potential utility of access to individualised chronic postsurgical pain risk profiles for clinical practice.

**Methods:**

This qualitative study included seven focus groups with 39 healthcare professionals from postanaesthesia care units, surgical units and orthopaedic wards across two hospitals in Norway. Data were analysed inductively using reflexive thematic analysis.

**Results:**

Our analyses yielded two overarching themes: (1) Lack of fit of risk predictions and (2) potentials of knowing risk profiles. Participants questioned the applicability of chronic postsurgical pain predictions in the in-hospital settings, highlighting role boundaries, time constraints, and limited influence over long-term outcomes. However, they also identified the benefits of risk awareness, including improved clinical reflection, more cautious decision-making, and an enhanced potential for individualised treatment and care.

**Conclusion:**

Healthcare professionals expressed a balanced view of chronic postsurgical pain risk profiles, recognising both scepticism about them and their potential benefits. Effective implementation will require predictive validity, clear guidance, and cross-disciplinary collaboration. Education and training will be essential to support clinicians in interpreting and acting on risk information.

## Introduction

The prevalence of chronic postsurgical pain (CPSP) varies widely, with estimates ranging from 5 to 85% depending on surgery type [1]. Adoption of the ICD-11 definition, pain arising or worsening after surgery and persisting beyond 3 months [2], has called previous prevalence and incidence estimates into question [3]. However, most mean incidence rates for CPSP are still approximately 20% [4], and a recent European survey reported a 6-month mean incidence of moderate-to-severe CPSP of 10.5% [5], highlighting its clinical burden. CPSP is associated with disability, opioid dependence, reduced quality of life, increased healthcare utilisation, and a considerable economic burden [6]. Furthermore, the management of CPSP is poorly documented and remains uncertain [7]. Risk factors extend beyond surgical variables to include various presurgical (e.g. pain, psychological comorbidity, health status, genetic predisposition) and postoperative factors (e.g. anxiety, depression, acute pain, and the pain trajectory) [1].

Increased attention has been given to the individualisation of perioperative pain management based on patient-specific risk predictions, with the aim of reducing the incidence of CPSP over time [7–9]. Some models and tools have demonstrated the potential to identify patients at risk of developing CPSP [4, 10, 11], and artificial intelligence (AI)—machine learning (ML) models have shown the potential to predict pain outcomes and prognostic trajectories [12, 13]. However, no prognostic models have demonstrated sufficient predictive accuracy or specificity for widespread clinical implementation [4, 12, 14]. A recent systematic review and meta-analysis [4] recommends including predictors from the entire perioperative period considering all potential risk factors.

Knowledge of individual risk may contribute to prevention and personalised treatment strategies [15–17]. However, there is limited understanding of how predictive risk information can be effectively integrated into routine clinical practice [18]. Some threshold risk predictors that are available at the point of care have demonstrated the ability to guide clinical decisions and improve CPSP outcomes [10], and personalised treatment has been proposed after risk identification [19, 20]. Yet, important questions persist regarding their overall clinical impact and the extent to which identified risk factors can be modified [14].

Research has examined healthcare professionals’ perspectives of clinical prediction rules [21] and the use of ML risk prediction models (for diseases or health conditions) in general [22]. However, little is known about how healthcare professionals perceive and interpret ML-predicted CPSP risk scores in perioperative care. Understanding the clinical work processes in which a tool would operate is essential [22, 23], since the working mechanism is to influence these processes to improve patient outcomes. Early assessment of context and stakeholder engagement during development and implementation are vital to ensure relevance [24], with qualitative feasibility studies offering valuable insights into perspectives and contextual factors affecting adoption [25]. Thus, this qualitative study aimed to explore the perspectives of in-hospital healthcare professionals and the potential relevance and utility of access to individualised risk profiles for CPSP in clinical practice.

## Methods

### Context

This research is part of a larger qualitative study (MAPIP-FEAS) that evaluates the clinical feasibility of continuous patient-reported pain monitoring to prevent CPSP. MAPIP-FEAS is linked to the MAPIP project, which develops a ML-based digital risk assessment system for CPSP based on patient-reported outcomes and continuous digital pain monitoring (REK 494978). Participants in the feasibility study were aware that the system was under development and had not yet begun clinical use. Part of the present dataset was analysed for a separate paper focusing on the perceived value of acute postoperative pain monitoring [26]. This current paper reports analyses of the perceived value of having access to risk profiles for CPSP. Collectively, these papers inform the development and implementation of the digital CPSP risk prediction system. The ‘Methods’ section in both papers will align [26]. The term *risk profiles* refer to individual risk scores potentially identified by the risk assessment system, allowing threshold-based classification of patients into different risk categories (e.g. high or low).

### Participants and recruitment

Participants were recruited from six departments across two hospital trusts in Western Norway and organised as focus groups. Recruitment followed participants’ responsibilities and involvement in perioperative pain management within the study hospitals. Most procedures were outpatient surgeries with short recovery periods in the postanaesthesia care unit (PACU). Patients with comorbidities or those undergoing more extensive surgery requiring inpatient care were managed in the intensive care unit (ICU) before transfer to the ward, usually within 24 h. In this setting, nurses assumed many pain-management responsibilities typically held by anaesthesiologists. Perioperative care involved one group of nurses conducting most preoperative assessments and another group managing the majority of postoperative pain, with both groups initiating consultation with physicians when necessary. Pain-management plans at discharge from the PACU or ICU were prescribed by the surgeon, while long-term follow-up at home was overseen by general practitioners and, if necessary, the surgeons. The focus groups included healthcare personnel working together during daily clinical practice, consisting of general surgeons, orthopaedic surgeons, and nurses. Anaesthetists were invited to participate but were unable to attend due to clinical demands. Recruitment was conducted through departmental information sessions, posters, and contact with departmental nurse managers [26]. Participants’ characteristics are presented in Table 1.

**Table 1:** Characteristics of the participants

		(*N* = 39)
Sex	Male	4
	Female	35
Age (years)	< 30	12
	30–39	11
	40–49	7
	≥50	9
Experience (years)	< 5	13
	5–14	12
	15–24	6
	≥25	8
Profession	AN	1
	RN	23
	ICU RN	2
	ICN	10
	Surg.	3

*AN* assistant nurse, *RN* registered nurse, *ICU RN* intensive care unit registered nurse, *ICN* ntensive care nurse, *Surg* general surgeon and orthopaedic surgeon

### Data collection

A semi-structured interview guide was developed through a combination of literature review, previous research, implementation theory, professional experience, and team discussions. The interview guide is available in Øvrebotten et al. [26] We conducted a pilot focus group and refined the guide for clarity. Feedback indicated that the flexibility in our interview guide fostered effective conversation. The pilot data were excluded from the analysis. Seven focus group interviews [range 57–120 min], totalling 495 min of audio recordings, were conducted between September and November 2023 and took place in the hospital where the participants were employed. The interviews were moderated by the first author (C. M. Ø.) and a co-moderator, alternating between RTH and a researcher (nurse anaesthetist) not co-authoring this paper. All moderators had prior experience conducting focus group interviews, and all focus group interviews were conducted in Norwegian. The interviews’ audio recordings were transcribed in Norwegian for the analyses.

### Data analysis

The data were analysed inductively following the six phases of Braun and Clarke’s reflexive thematic analysis [27]. First, all authors (C. M. Ø.: nurse, paramedic, and PhD student; RTH: social scientist and associate professor; SBB: operating room nurse and professor; and CM: specialist psychologist and professor) read the transcribed interviews and shared initial impressions during the first analysis meeting. We believe that the interdisciplinary team enriched the discussions by incorporating a diverse range of perspectives. In reflexive thematic analysis, researcher subjectivity is a resource when critically reflected upon, acknowledging that knowledge is contextually produced [27]. Second, the first author conducted descriptive coding in NVivo and organised the codes into groups. Third, preliminary themes were developed by the first author and discussed within the team toward collaborative refinement and further development. Themes were then visualised using thematic maps and revised through repeated discussions, in particular between the first and last authors. Thereafter, the write-up process contributed to further analytical refinement, reflecting the dynamic and reflexive nature of the approach. Finally, the data were translated to English after the analyses were complete. For more details on the analytical process, see Øvrebotten and colleagues [26]. The coding tree (Table 2) provides a visual representation of how the coding process relates to the sub-themes and main theme.

**Table 2:**
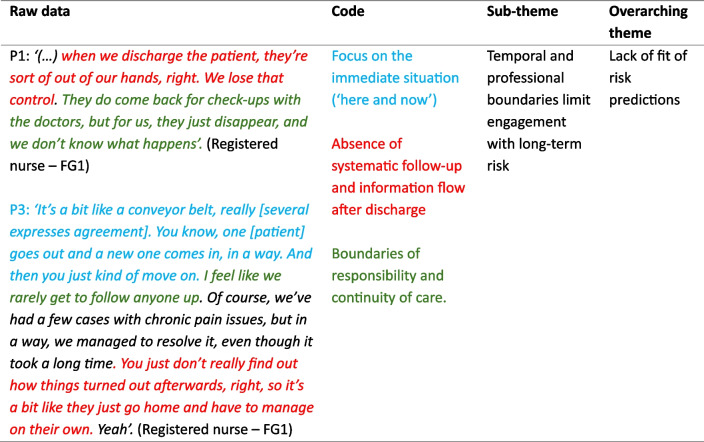
Example of a coding tree

### Ethical considerations

The study was reviewed by the Data Protection Officer at Førde Hospital Trust (eProtocol 3942) and partner hospital trusts obtained approval from their local data protection officers. The Regional Committee for Medical and Health Research Ethics in Western Norway exempted the project from further ethical committee review. Informed consent was obtained from all participants. All data were anonymised and stored securely.

## Results

**Fig. 1:**
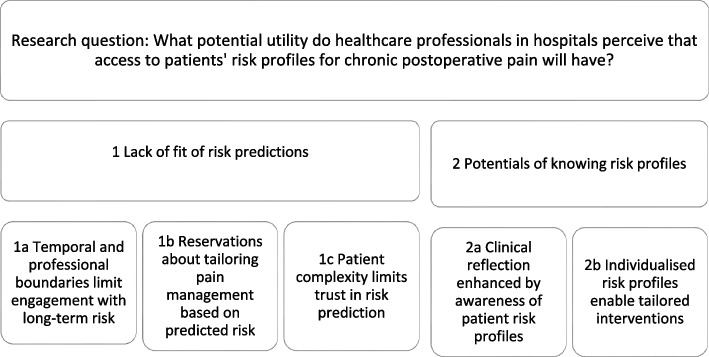
Overview of the overarching themes and sub-themes

The analysis resulted in two overarching themes, each with associated sub-themes: (1) *Lack of fit of risk predictions* and (2) *Potentials of knowing risk profiles* (illustrated in Fig. 1). These themes reflect the spectrum of scepticism and perceived benefit among the participants. Quotes illustrate the participants’ (P) perspectives. We use (…) within quotes to indicate that extraneous text has been removed for clarity. Table 3 details sub-themes and illustrates which focus groups contributed to which theme.

**Table 3:** Source matrix presenting focus groups involved in the themes and sub-themes

Focus groups	FG1	FG2	FG3	FG4*	FG5*	FG6	FG7*
Number of participants (*N* = 39)	7	5	8	4	4	7	4
Overarching themes and sub-themes							
1. Lack of fit of risk predictions	X		X	X	X	X	X
1a. Temporal and professional boundaries limit engagement with long-term risk	X		X		X	X	X
1b. Reservations about tailoring pain management based on predicted risk	X			X	X	X	
1c. Patient complexity limits trust in risk prediction	X		X			X	
2. Potentials of knowing risk profiles	X	X		X	X	X	X
2a. Clinical reflection enhanced by awareness of patient risk profiles	X			X	X	X	X
2b. Individualised risk profiles enable tailored interventions		X		X	X	X	X

*FG *focus group. *Postanaesthesia care unit. Table legend: FG1, FG5, and FG6 contributed to most sub-themes, suggesting broad engagement with both challenges and opportunities. FG3 contributed only to Theme 1 and FG2 only to Theme 2, possibly reflecting group-specific experiences. While some sub-themes were discussed by only three or four groups, they still yielded valuable insights. Sub-theme 1c was relevant mainly to regular departments, not postanaesthesia care units. Overall, six of the seven focus groups were well represented across the overarching themes

### Overarching Theme 1—Lack of fit of risk predictions

Participants expressed scepticism about the clinical utility of CPSP risk profiles. Their concerns underscored the opinion that CPSP risk predictions remain poorly aligned with clinical realities. Three sub-themes comprised this overarching theme.

#### 1a. Temporal and professional boundaries limit engagement with long-term risk

This sub-theme focused on how participants oriented their attention towards the immediate postoperative phase, rather than long-term patient outcomes. It highlighted how participants viewed their responsibility as confined to the hospital stay, with patient care considered complete upon discharge.

Participants described an acute care mindset, where long-term consequences such as chronic pain were perceived as distant, abstract, or outside their professional remit.

P27: I don’t really think much about preventing chronic pain, because it’s more about the’ here and now’, and I don’t really see the longer-term perspective. (…) The most important thing is, of course, the immediate experience, that the patient shouldn’t be lying there in pain. (ICN – FG5)

Another key aspect was the professional boundary between hospital-based care and post-discharge management. There was a consensus among the participants that their treatment focus was limited to acute care; P32: ‘We treat them here and now, not outside [the hospital] in a way (…)’ [Several express agreements] (RN—FG6). Their professional responsibility was seen to end at discharge, with little opportunity for follow-up or feedback on patient progress. As one participant noted, P1: ‘(…) when we discharge the patient, they’re sort of out of our hands, right? We lose that control. They do come back for check-ups with the doctors, but for us, they just disappear, and we don’t know what happens’ (RN—FG1).

Participants also noted professional boundaries between departments, with acute care teams viewing long-term management as the responsibility of others; P39: ‘We [at the post-anaesthesia care unit] are in a bit of a special position really, because we often see them when they’re at their worst and when we have to give a lot [of analgesics]’ (ICN—FG7).

In contrast to this acute focus, some participants recognised their potential role in influencing long-term outcomes; P13: ‘We orthopaedic surgeons pose a significant risk to the patients, as they may end up in a situation of dependency and chronic pain’ (Surg.—FG3). A few described how optimal perioperative pain management could potentially prevent chronic pain in high-risk populations, yet this knowledge was not systematically applied; P26: ‘When it comes to amputations and so on… if they’re well managed both before and after surgery, it prevents phantom pain, doesn’t it? So that’s important’ (ICN—FG5).

In summary, these findings illustrate how a short-term clinical horizon—reinforced by institutional departmentalisation, professional role boundaries, and a lack of outcome visibility—limited the potential for early preventive action. Even when healthcare professionals recognise long-term risks, their ability to act on their awareness remains constrained by a system that favours the ‘here and now’.

#### 1b. Reservations about tailoring pain management based on predicted risk

Participants expressed hesitation about using predicted CPSP risk to guide treatment decisions. The participants were uncomfortable with the idea of increasing or withholding analgesia based on a patient’s presumed risk; P27: ‘I wouldn’t give someone more pain relief [knowing their CPSP risk—laughs a little]. I mean, ‘He can have a bit more pain because he’s not at risk for developing chronic pain’—I wouldn’t think like that (ICN—FG5). Some raised ethical concerns, emphasising that all patients should receive adequate pain relief regardless of predicted risk. One participant described how differentiating care based on risk profiles felt wrong; P34: ‘We’re not supposed to do that. (…) We’re supposed to treat everyone the same …’ (RN—FG6). This core ethical stance that all patients should receive adequate pain relief regardless of risk status was clearly expressed by one participant; P22: ‘Well, we want to treat pain as best we can…’ (ICN—FG4).

Participants discussed unintended consequences of informing patients they were ‘high risk’, suggesting this might amplify anxiety or pain; P3: ‘If the patient knows they’re at high risk of developing pain postoperatively, that might not necessarily be positive. If they go in thinking, ‘oh no, this is going to hurt’- then it will probably hurt more’ (RN—FG1). They acknowledged, however, that reactions would likely vary between individuals; P2: ‘(…) it depends entirely on the person’ (RN—FG1). 

#### 1c. Patient complexity limits trust in risk prediction

This sub-theme reflects scepticism about the possibility of accurately predicting CPSP development. Participants questioned the premise that risk could be static or easily classifiable. Participants in one focus group reflected on the limitations of a binary low/high risk framework; P1: ‘If someone is labelled low risk, could they still actually be high risk in some way? Is it really that black and white?’ (RN—FG1). As underscored by this quote, P5: ‘There’s no definitive answer. There’s no guarantee it’s correct’ (RN—FG1). 

Participants emphasised that risk status may shift over time, illustrating resistance to simplified assumptions about pain trajectories, viewing postoperative pain development as uncertain and influenced by multiple evolving factors; P31: ‘Just because they were high risk before doesn’t mean it’ll be the same this time’. 

The perceived limitations of prediction were further linked to concerns about clinical validity. A key concern was that some tools might be perceived as too generic or disconnected from the patient’s actual, current context. A surgeon explained the following:

P13: But we have so many different groups of patients. What kind of tool… yeah, it would be exciting to have a tool that could tell us that “here we have someone that ends up” … But then there’s the individual situation, the psychosocial situation, which matters a lot – and then there’s the backpack they’re carrying with them. (Surg. - FG3)

Overall, participants viewed risk as dynamic, context dependent, and difficult to pin down. While some saw potential in prediction tools, scepticism remained about whether a profile could adequately capture the nuances of individual patients and shifting clinical conditions.

### Overarching Theme 2—Potentials of knowing risk profiles

Participants discussed the potential benefits of having access to patient-specific CPSP risk information, e.g. sharpened clinical judgement, reflection, and more targeted and individualised care. This overarching theme included two sub-themes: (2a) *Clinical reflection enhanced by awareness of patient risk profiles* and (2b) *Individualised risk profiles enable tailored interventions.*

#### 2a. Clinical reflection enhanced by awareness of patient risk profiles

Some participants described how information about CPSP risk factors challenged their existing understanding and heightened awareness of their own clinical practice:

P2: (…) I was surprised when you [the moderator] talked to us the first time, and mentioned how important those first few days [after surgery] are, considering that they [surgical patients] can develop chronic [pain]. (…) That hasn’t been on my mind, it really hasn’t. (RN – FG1)

Some said they already gather patient information in current practice, and that certain ‘red flags’ lead to a more cautious approach. They said risk prediction would likely activate similar awareness as follows:

P24: (…) if we had that information [risk profile] in addition (…) it would be useful for how I can approach this patient. To have a bit of a plan already in my head, about what might be a good way to meet this patient and. (ICN – FG4)

Others described how this knowledge could enhance professional judgement. This might prompt them to pause and think before acting, as expressed by one ICN; P36: ‘I would imagine that [knowledge about high risk] would influence how we think. You’d probably be more cautious about pain management, think things through more’ [several express agreements] (ICN – FG7).

This recognition of the need to challenge established patterns of thought and think more long term in pain management also emerged in reflections about how inadequate or short-sighted treatment in the acute phase could lead to more complicated pain trajectories.

P30: I think that any information you can get about the patient beforehand might help improve the course. (…). You should keep in mind that they might face challenges later that make pain relief harder next time (…) [several express agreements]. (RN – FG6)

This sub-theme reflects cognitive alertness rather than specific actions, where information about risk could lead them to become more aware and increase their preparedness for interaction with patients.

#### 2b. Individualised risk profiles enable tailored interventions

This sub-theme focused on how access to predictive risk profiles may directly support clinical decision-making and enable early, individualised interventions. The participants described how risk information could influence key treatment decisions, including whether to proceed with surgery in high-risk patients; P11: ‘(…) if you can avoid operating on the patient, then the recommendation is not to operate, for example because you risk worsening the condition’ (Surg. – FG2). However, they also acknowledged the complexity of such decisions. The risk of chronic pain needed to be weighed against potential quality-of-life improvements is as follows:

P10: (…) if they’re in so much pain in one knee that they can’t walk at all and have poor quality of life, then some get much better in that knee after surgery. But then, there’s a different kind of risk – yes, chronic pain. Yes, it’s … “the plague or cholera”. (RN – FG2)

Some participants reflected on previous experiences in clinical practice and the opportunities to access risk profiles, which could be utilised for initiating more advanced interventions early on to improve treatment. For example, participants described specific interventions they would consider if they had foreknowledge of high risk; P30: ‘If we had had info in Meona [the digital journal]: “This patient is at high risk for developing chronic pain afterwards” (…), then we could have placed an epidural, for instance’ (RN—FG6).

Some participants emphasised that risk profiles could inform medication choices in terms of both advanced pharmacological interventions and tailored support for vulnerable groups, such as those with anxiety, depression, prior opioid use, or pain following limb amputations.

P27: But that you then [with risk prediction] might be thinking ahead about other things too. Like with amputations – we sometimes talk about Tegretol and some of the others, Neurontin or GABA inhibitors… I often feel that kind of thing comes too late in the system, not in the immediate phase. It would’ve been smart to get that in earlier. (ICN– FG5)

Participants described how risk information could legitimise or enable more individually tailored decisions, both among nurses and physicians.

P36: Because they usually just provide this “preset” package [standard analgesics, preconfigured in the electronic prescribing system] and say, “You have to make this work.’ But then maybe they’d be a bit more cautious with certain medications, or suggest different types, or… So that might be a positive thing in that case. (ICN – FG7)

Overall, this sub-theme underscored how risk information can support more proactive and tailored clinical actions. Rather than primarily influencing attitudes or awareness, this sub-theme illustrates how predictive knowledge can inform concrete decisions and enable early, individualised interventions.

## Discussion

This study explored healthcare professionals’ perceptions of the implementation of risk predictions for CPSP as part of perioperative care because early stakeholder involvement is vital in ML model design and implementation [22]. We found scepticism about risk prediction; it was perceived to be poorly aligned with clinical realities and, therefore, perceived as challenging to integrate into practice. Participants also saw potential in patient-specific CPSP risk information to support reflection and clinical judgement and more personalised care. This suggests that the same information may be regarded as both redundant and valuable, depending on its integration into practice.

### The utility of predicted risk depends on the credibility of the tool

Healthcare professionals in our study expressed concerns about acting on CPSP risk profiles. This involved ethical concerns about making clinical decisions based on predictions generated by ML tools [28], whose evidential validity for clinical use in healthcare across different settings and patient groups remains limited [9, 29]. For healthcare professionals to consider them to be trustworthy, evidence that risk profiles make accurate and reliable risk predictions across different clinical contexts seems to be crucial [12, 30].

Our participants warned that awareness of high risk could have serious consequences, such as exclusion from surgery. Similar concerns include fear of the unintended consequences of AI and accountability when predictors misalign with clinical observations, which have been reported elsewhere [22, 31]. These findings underscore the need to address ethical considerations in implementation. The fear of exposing patients to negative outcomes due to AI predictions is also one of the most frequently reported barriers in the literature [32]. ML/AI-generated solutions must reflect patients’ actual needs and safeguard autonomy [22]. Risk prediction can support perioperative decision-making, but it requires both accurate estimations and the availability of effective interventions tailored to risk groups [30]. Specific guidance on how to manage predicted risk is needed to ensure that clinical judgement remains central. In summary, CPSP risk predictions are useful only when clinicians trust the tool’s credibility and validation.

### Limited utility when information pertains to other stages of the care pathway

Participants defined their responsibilities narrowly, particularly regarding follow-up after discharge, which they considered to be other providers’ responsibility. Our findings also revealed divisions between hospital departments among staff encountering patients at different stages of the pain trajectory. Such organisational silos foster shared routines and inward-facing practices that limit information flow and interdisciplinary collaboration, thereby constraining continuity of care [33]. Participants noted that these structures reduced opportunities for CPSP prevention, as their clinical responsibilities were largely restricted to acute concerns. Consequently, risk predictions were perceived to have limited relevance when they addressed stages of the patient pathway beyond the immediate in-hospital encounter. Fragmentation and inadequate coordination both hinder effective pain management [34] and implementation of new solutions [35]. In our context, fragmentation risks leaving patients with evolving pain trajectories that are insufficiently followed up, thereby potentially increasing the likelihood of CPSP. The literature emphasises the need for digital tools such as ML/AI-generated solutions to integrate them into existing workflows and clinical processes [12, 22, 26]. However, our findings suggest that when a tool is intended to span the entire perioperative pathway, it requires a broader conceptualisation of professional roles, extending attention beyond the immediate clinical setting.

Participants paid little attention to CPSP prevention, but they did not consider it irrelevant. It was viewed as having limited applicability when it fell outside the acute clinical remission of acute pain management and opioid-related concerns. These ambiguities about responsibility and timing of interventions for CPSP echoed the research literature [15, 16]. Addressing CPSP is likely to require closer interdisciplinary collaboration across perioperative teams [15], and given the number of actors involved in perioperative care, strengthening collaboration across teams is essential [36]. Anaesthesiologists are potential leaders of structured, multidisciplinary perioperative teams to reduce CPSP. However, the utility of risk predictions depends on a shared understanding of their application throughout the patient pathway prioritising teamwork across professions, departments, and levels of care [15, 17, 34]. This requires systems that facilitate cross-boundary communication and coordination [37], as well as cultivating a shared sense of responsibility for CPSP prevention [16]. Such an approach entails a cultural shift, emphasising integrative practice, role clarity, and collective goals rather than narrow self-interest [33, 37]. Without a shared understanding of how risk predictions should be applied at different points in the patient pathway [16], their utility is likely to be diminished.

### Predicted risk requires a different type of clinical reflection

Our findings show that participants recognised the potential of CPSP risk predictions to support more personalised and proactive interventions, in line with literature emphasising the importance of risk-informed prevention [16]. Participants described knowledge gaps, particularly regarding how complex pain trajectories contribute to CPSP risk, and the need for more targeted measures during hospitalisation and during the subacute phase [38]. Talking about CPSP risk made participants more aware of both their preventive role and their own knowledge gaps, a finding consistent with previous literature [35]. A previously noted challenge is the lack of standardised practice regarding when and how interventions to prevent CPSP should be initiated [14, 34]. Knowledge must be embedded into organisational and clinical routines to enhance the utility of risk prediction [36]. As routines are enacted and adapted by individuals who think and reflect, they hold substantial potential for change and development [39]. This highlights the need for clear action plans and guidelines to support healthcare professionals across the entire pain trajectory and ensure continuity of follow-up.

Education and training will therefore be essential to foster acceptance and awareness of the importance of integrated and multidisciplinary perioperative care [17]. Structured plans should ensure that high CPSP risk patients are followed before, during, and after hospitalisation, requiring multidisciplinary perioperative teams that collaborate across departments to improve outcomes [15]. In summary, this indicates that CPSP risk predictions can only add value if clinicians are supported with the knowledge, reflection, and guidance necessary to interpret and act on them across the full perioperative pathway.

### Strengths and limitations

Although some aspects of the methods and the discussion of the study’s strengths and limitations resemble our previous publication [26], as both articles are based on the same dataset and study context, the present article focuses on a different research question and provides new, independent analyses and findings. In accordance with research ethics guidelines, we openly acknowledge this relationship to ensure transparency in reporting. The detailed methodological description and contextual framing enable readers to assess the study’s relevance for other settings, in line with qualitative principles of transferability [40]. A key strength of this study is its rich and varied data, derived from focus group interviews with 39 healthcare professionals across 6 departments, including nurses, specialist nurses, surgeons, and orthopaedic surgeons. This interdisciplinary composition enabled a nuanced exploration of perspectives on the potential utility of CPSP risk profiles in clinical practice. The limitations include challenges in recruiting anaesthesiologists and surgeons, which may have narrowed the range of professional perspectives. A key limitation of this study is the absence of anaesthesiologists in the interview groups. However, this limitation is partly mitigated by the study context: many pain-management responsibilities typically held by anaesthesiologists were delegated to nurses or shared between nurses and anaesthesiologists. The short timeframe of responsibility described by the nurses in this study was likewise shared by anaesthesiologists, while general practitioners oversaw long-term pain management consulting with surgeons when necessary. For CPSP and long-term outcomes, coordinated pain-management planning and follow-up by the surgical departments are essential. A strength of the study is therefore the inclusion of physicians from both general surgery and orthopaedic surgery. Another mitigation for the lack of anaesthesiologist participant was the inclusion of one senior anaesthesiologist as team member and co-author, responsible for reviewing the context, data, and analysis process and considering limitations of our approach for reader transparency. Conducting the study at two Norwegian hospitals may have limited transferability to other countries, where healthcare systems, professional roles, and education differ. Recruitment via department nurse leaders may have influenced voluntariness, though this was mitigated through clear communication and written informed consent. The discussion of risk profiles occurred at the end of the interviews, potentially affecting participants’ focus and depth of reflection. As risk prediction tools were not yet implemented, participants engaged with verbal descriptions rather than actual tools. Although more pre-information might have aided understanding, limiting details helped avoid biasing responses. Despite these limitations, the data are sufficiently rich to address the research question meaningfully.

## Conclusion

Our findings reveal both scepticism about and recognition of the potential benefits of CPSP risk prediction. The challenges lie in establishing credibility, embedding predictions across fragmented pathways, and fostering reflective practice. Addressing these requires rigorous validation, clear guidance, and multidisciplinary collaboration. Education and organisational change will be essential for enabling clinicians to apply risk predictions in a way that enhances risk-informed prevention and patient-centred perioperative care.

## Data Availability

Due to ethical and privacy considerations, the dataset cannot be shared. As this was a qualitative study, only anonymised excerpts are included in the manuscript. Quotations are attributed using numerical identifiers and role descriptions to ensure confidentiality. Any requests for additional information may be directed to the corresponding author.

## Abbreviations

AI: Artificial intelligence
AN: Assistant nurse
CPSP: Chronic postsurgical pain
FG: Focus group
ICN: Intensive care nurse
ICU RN: Intensive care unit registered nurse
ML: Machine learning
RN: Registered nurse
Surg.: General surgeon/orthopaedic surgeon
